# The problem of obesity among adolescents in Hong Kong: a comparison using various diagnostic criteria

**DOI:** 10.1186/1471-2431-8-10

**Published:** 2008-03-04

**Authors:** Gary TC Ko, Risa Ozaki, Gary WK Wong, Alice PS Kong, Wing-Yee So, Peter CY Tong, Michael HM Chan, Chung-Shun Ho, Christopher WK Lam, Juliana CN Chan

**Affiliations:** 1 The Hong Kong Institute of Diabetes and Obesity, Hong Kong SAR, China; 2 Department of Medicine and Therapeutics, The Chinese University of Hong Kong, Prince of Wales Hospital, Shatin, Hong Kong SAR, China; 3 Department of Pediatrics, The Chinese University of Hong Kong, Prince of Wales Hospital, Shatin, Hong Kong SAR, China; 4 Department of Chemical Pathology, The Chinese University of Hong Kong, Prince of Wales Hospital, Shatin, Hong Kong SAR, China

## Abstract

**Background:**

Obesity is now a global epidemic. In this study, we aimed to assess the rates of obesity using several major diagnostic criteria in Chinese school adolescents in Hong Kong.

**Methods:**

This is a cross-sectional study. Using a computer-generated coding system, we randomly selected schools from different geographical regions in Hong Kong to obtain a representative sample. Subjects aged 11–18 years of age were randomly selected from different class of the schools. Their rates of obesity according to four different international and local criteria were compared [International Obesity Task Force (IOTF) 2000 criterion; the Group of China Obesity Task Force (COTF) 2004 criterion; Centers for Disease Control and Prevention (CDC) 2000 Growth Charts and the Hong Kong Growth Survey (HKGS) charts in 1993].

**Results:**

Of the 2098 adolescents [982 (46.8%) boys and 1116 (53.2%) girls], the mean age (± SD) was 15.1 ± 1.8 years (range: 11–18 years; median: 15.0 years). The crude rates of obesity were similar based on IOTF, COTF or CDC criteria (boys: 3.9–6.0%, girls: 1.8–3.7%), however, the rate increased to 11–27% if the HKGS charts were used. Obesity rate varied markedly according to age. It decreased from 8–10% among those aged 12–13 years to 2–4% among those aged 17–18 years.

**Conclusion:**

The prevalence of obesity in Hong Kong adolescents using various diagnostic criteria were similar except for the 1993 HKGS criteria, which gave an exceeding high figure. Using the IOTF, COTF or CDC criteria, the adolescent obesity in Hong Kong varied from 1.8% to 6.0%.

## Background

Obesity is now a global concern not only in adults but also among children and adolescents [1]. There is now consensus on the negative impact of obesity on physical, mental and social functions in children [2,3]. Moreover, many obese children remain obese in their adulthood [4], with possible increased risk of adult mortality and morbidity [5-7].

In 2005, World Health Organization estimated that more than 20 million children above age of 5 were overweight worldwide [8]. Alongside with the rising prevalence of childhood obesity in Western countries, the same phenomenon is also running rampant in our region. In the Chinese National Survey on Students Constitution and Health conducted in 2000, using BMI values of 24 kg/m^2 ^and 28 kg/m^2 ^as cutoff points for overweight and obesity respectively (the definition by the Working Group on Obesity in China) [9], the corresponding prevalence in Chinese children aged 7–18 years were 17.0% and 10.0% for boys and 9.5% and 6.5% for girls, respectively [10].

Despite increasing number of publications on childhood obesity in the Western countries, similar information in Hong Kong children remains scanty. Besides, there are ongoing debate on the most 'appropriate' definition and optimal cutoff values for childhood obesity and overweight with different diagnostic criteria adopted by different countries and authorities [11-14]. In this survey, we examined the rates of overweight and obesity in 2098 Hong Kong Chinese school adolescents aged 11–18 years using several major diagnostic criteria and their variability. We aimed to report the problem of obesity in Hong Kong adolescents and compare their rates according to various diagnostic criteria. We also attempted to explore the reasons and significance behind the difference in obesity rates by these criteria. This will help us to better understand them and improve the modification of our diagnostic criteria in the future if deemed necessary.

## Methods

A full list of all Chinese secondary schools in Hong Kong was obtained from the Hong Kong Education Department. Hong Kong comprises 3 major geographical regions including Hong Kong Island, Kowloon and the New Territories with a population of 6.7 million.

Based on the full list of Chinese secondary schools in Hong Kong, we randomly selected schools from each of the 3 geographical regions to obtain a representative sample population of Hong Kong Chinese adolescents. The randomization was based on a computer-generated coding system. Amongst a total of 477 schools, 53 schools (10% of the total) were selected. From each participating school, 6 classes were randomly selected with one class from each form (Form 1 to Form 6) to obtain a proportional number of subjects aged 11–18 years of age. All participants gave informed consent with parents' written consent.

Our team of research nurses visited each school during an allocated time in the morning for measurement of anthropometric indices. Body weight (kg) and body height (m) were measured and the body mass index (BMI) was calculated. All data were collected during the period between February 2003 and February 2004.

### Definitions of overweight and obesity

Four criteria were used for the definitions of overweight and obesity:

1. An international BMI-for-age reference curve for defining overweight and obesity in children 2 to 18 years of age by the US National Center for Health Statistics, Centers for Disease Control and Prevention (CDC) and the International Obesity Task Force (IOTF) in 2000 (IOTF criteria) [11] -

These criteria were based on median BMI by age and gender in six nationally representative datasets from Brazil, Hong Kong, Netherlands, Singapore, the UK and the US from an international growth survey in 2000. These surveys had over 10,000 subjects each and together covered 97,876 boys and 94,841 girls. Overweight and obesity were defined as BMI-for-age ≥ 25 and ≥ 30 kg/m^2 ^respectively.

2. A national BMI reference curve for Chinese children and adolescents reported by the Group of China Obesity Task Force (COTF) in 2004 (COTF criteria) [10] -

These criteria were based on the Chinese National Survey on Students Constitution and Health in 2000 involving 244,200 primary and secondary Chinese students aged 7–18 years. Overweight and obesity were defined as BMI-for-age ≥ 24 and ≥ 28 kg/m^2 ^respectively.

3. CDC 2000 Growth Charts for the United States (CDC criteria) [15] -

These criteria were based on the US National data collected in a series of 5 surveys between 1963 and 1994 for children and adolescents aged 2–20 years. Overweight and obesity were defined as BMI-for-age ≥ 85 and ≥ 95 percentiles respectively.

4. The Hong Kong Growth Survey (HKGS) conducted in 1993 with sex-specific reference charts of weight-for-height (HKGS criteria) [16] -

This is the only local reference used to define obesity in Hong Kong children. This was a territory-wide cross-sectional growth survey which covered around 25,000 Hong Kong Chinese children from birth to 18 years of age. Childhood obesity in this survey was defined as weight > median weight for height × 120%. No definition for childhood overweight was set in this survey.

### Statistical analysis

Statistical analysis was performed using the Statistical Package for Social Science software (version 12.0). All data are expressed as mean ± SD or n (%) where appropriate. Chi-square tests, ANOVA and Student's t test were used for group comparisons. All comparisons were made two-sided and a *p*-value < 0.05 (2-tailed) was considered as significant.

### Ethical Approval

The study was approved by the Clinical Research Ethics Committee of the Chinese University of Hong Kong.

## Results

Of the 53 schools selected, 14 schools consented and were recruited for the survey. From these 14 schools, random samples of 4598 students were identified from their six forms (1 to 2 classes were randomly selected from each form of each school with 30 to 45 students per each class). Of these, 2115 school children consented and were enrolled into the study giving a response rate of 46%. Of the 2115 children, 17 missed their anthropometric assessment. Hence, data on 2098 school children aged 11 to 18 years of age were analyzed in this survey. There were 982 (46.8%) boys and 1116 (53.2%) girls with a mean age (± SD) of 15.1 ± 1.8 years (range: 11–18 years; median: 15.0 years).

Table 1 summarizes the anthropometric parameters and rates of overweight and obesity by various diagnostic criteria in the 2098 Hong Kong Chinese adolescents. The mean crude prevalence of overweight and obesity varied from 9.8–13.9% and 2.7–15.8% respectively, according to different diagnostic criteria. Table 2 summarizes their anthropometric parameters and rates of overweight and obesity by age groups. Those aged 11 were discarded due to small sample size (boys: n = 9; girls: n = 11). The mean crude BMI varied from 19.1 to 21.1 kg/m^2 ^in boys and 17.9 to 20.2 kg/m^2 ^in girls.

**Table 1:** Clinical parameters and prevalence of overweight and obesity by various diagnostic criteria in 2098 Hong Kong Chinese adolescents.

	Total (n = 2098)	Boys (n = 982)	Girls (n = 1116)	p-values comparing boys and girls
Age, years	15.1 ± 1.8	14.9 ± 1.8	15.3 ± 1.8	< 0.001
Height, cm	161.4 ± 8.5	165.8 ± 9.2	157.7 ± 5.5	< 0.001
Weight, kg	52.3 ± 11.5	56.1 ± 13.0	48.9 ± 8.8	< 0.001
BMI, kg/m^2^	19.9 ± 3.5	20.3 ± 3.8	19.7 ± 3.2	< 0.001

Childhood overweight, %				
IOTF	9.91	13.54	6.72	< 0.001
COTF	14.06	19.25	9.50	< 0.001
CDC	12.44	17.31	8.15	< 0.001

Childhood obesity, %				
IOTF	2.76	3.87	1.79	0.004
COTF	4.77	6.01	3.67	0.012
CDC	4.10	6.01	2.42	< 0.001
HKGS	15.87	18.13	13.89	0.008

IOTF, International Obesity Task Force; COTF, China Obesity Task Force; CDC, Center of Disease Control; HKGS, Hong Kong Growth Survey

**Table 2:** Anthropometric parameters and prevalence of overweight and obesity of 2098 Hong Kong Chinese adolescents stratified by age and sex.

				Overweight			Obesity			
	Height, cm	Weight, kg	BMI, kg/m^2^	IOTF	COTF	CDC	IOTF	COTF	CDC	HKGS

Boys (n = 973):										
Age, years										
12 (n = 97)	154.4 ± 9.3	47.0 ± 11.4	19.5 ± 3.6	23.7	30.9	30.9	5.2	4.2	8.2	25.8
13 (n = 161)	158.7 ± 8.9	50.9 ± 13.9	20.0 ± 4.2	20.5	27.3	28.0	6.8	9.9	10.6	27.3
14 (n = 147)	165.3 ± 7.6	55.7 ± 14.1	20.2 ± 4.4	15.0	19.7	19.0	4.1	7.5	7.5	19.7
15 (n = 189)	168.3 ± 5.6	56.7 ± 11.4	20.0 ± 4.4	12.2	17.5	14.3	2.6	4.8	3.7	13.8
16 (n = 185)	170.2 ± 5.9	59.7 ± 11.1	20.0 ± 3.5	9.2	15.1	10.8	2.7	3.8	3.8	14.6
17 (n = 96)	171.3 ± 5.5	62.0 ± 10.8	21.1 ± 3.4	9.4	15.6	12.5	3.1	4.2	4.2	14.6
18 (n = 98)	172.0 ± 5.6	61.3 ± 11.1	20.7 ± 3.3	5.1	8.2	6.1	2.0	3.1	3.1	11.2

Girls (n = 1104):										
Age, years										

12 (n = 57)	152.8 ± 5.0	43.7 ± 8.1	18.7 ± 3.1	17.5	15.8	17.5	0	5.3	3.5	15.8
13 (n = 138)	155.7 ± 6.3	46.7 ± 9.5	19.2 ± 3.3	9.4	12.3	11.6	2.2	4.3	2.9	16.7
14 (n = 156)	157.3 ± 4.7	48.1 ± 8.6	19.4 ± 3.3	8.3	12.8	11.5	1.9	3.8	1.9	13.5
15 (n = 186)	158.5 ± 5.5	50.8 ± 9.6	20.2 ± 3.5	7.5	10.8	10.8	3.2	5.9	5.4	17.2
16 (n = 283)	158.6 ± 5.1	50.0 ± 8.4	19.8 ± 3.0	4.6	7.8	4.9	1.8	2.5	1.8	12.0
17 (n = 109)	157.8 ± 4.9	49.7 ± 8.6	19.9 ± 3.2	3.7	6.4	4.6	1.8	3.7	1.8	11.9
18 (n = 175)	158.8 ± 5.0	49.5 ± 7.4	19.6 ± 2.8	3.4	5.7	3.4	0.6	2.3	0.6	12.0

**Figure 1:**
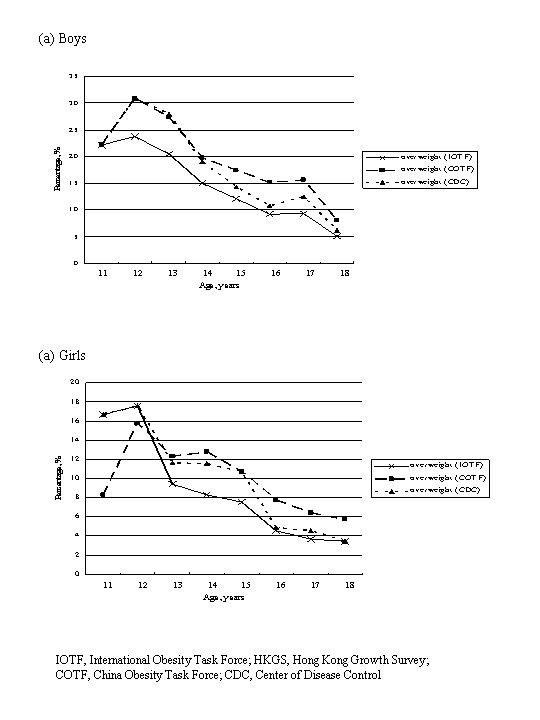
Percentages of overweight as defined by various criteria in 2098 Hong Kong Chinese adolescents categorized by age and gender.

**Figure 2:**
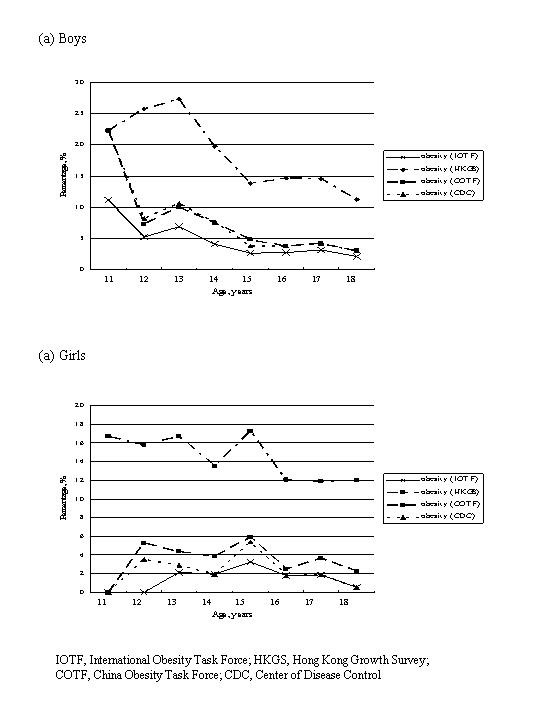
Percentages of obesity as defined by various criteria in 2098 Hong Kong Chinese adolescents categorized by age and gender.

Figures 1 and 2 summarize the rates of overweight and obesity as defined by various criteria in the 2098 Hong Kong Chinese adolescents categorized by age and gender. The IOTF, COTF and CDC criteria showed similar rates of overweight and obesity in Hong Kong Chinese adolescents in most age groups. Based on these 3 criteria, the rates of overweight varied from 5–8% in those aged 18 years to 24–31% in those aged 12 years among boys; and 4–6% in those aged 18 years to 16–18% in those aged 12 years among girls, while the rates of obesity varied from around 3% in those aged 18 years to 11–22% in those aged 11 years among boys; and 1–2% in those aged 18 years to 0–6% in those aged 12 years among girls. The prevalence of obesity was much higher in boys than girls (crude overall obesity rates: 3.9–6.0% vs. 1.8–3.7%, depending on different diagnostic criteria).

The rates of obesity based on the HKGS criteria were much higher than those derived from the IOTF, COTF or CDC criteria (crude overall obesity rates: 18.1% in boys and 13.9% in girls). The rates of obesity varied from 11% in those aged 18 years to 22–27% in those aged 11–13 years among boys; and 12% in those aged 18 years to 16–17% in those aged 11–13 years among girls.

## Discussion

Our study was limited by the volunteer nature of the respondents. Nevertheless, the response rate was approximately 50% which is comparable with most volunteer surveys. In this survey, we have noted high prevalence of obesity in Hong Kong adolescents with good consistencies amongst international and China definitions. In Hong Kong, all children are required by law to receive formal school education up to Form 3 (equivalent to Year 9 in the United States) with the majority completing Form 5 education. Hence, a sample from school children should be able to represent the overall young populations in Hong Kong. The random recruitment method in this survey of children from all secondary schools in Hong Kong also optimized the representative nature of our samples. However, the relatively small sample size of individual age group in our survey, especially among those aged 11 years, may introduce potential bias. Unfortunately, out of the 53 schools that were selected, only 14 agreed to participate. Although we cannot access the students' clinical particulars among those schools that refused to join our study, according to the full list of secondary schools in Hong Kong, the schools being recruited were evenly distributed in different regions in Hong Kong across a wide range of socio-economic classes.

Obesity is associated with significant morbidity and mortality [17]. There is now growing concerns on the increasing prevalence in childhood obesity and most obese children will grow up to become obese adults and most obesity related health problems are also applicable to children [2-7]. Despite the global epidemic of childhood obesity and associated health burden, the most 'appropriate' criterion to diagnose obesity in children is still inconclusive [11-14].

In the year 2000, an international BMI-for-age reference curve for defining overweight and obesity in children 2 to 18 years of age was developed jointly by the US National Center for Health Statistics, Centers for Disease Control and Prevention and the IOTF (IOTF criteria) [11]. The reference population was obtained from 6 large nationally representative cross-sectional growth surveys in the US, the UK, the Netherlands, Brazil, Hong Kong and Singapore which collectively involved close to 200,000 young subjects. On a national basis, both China and the US have recommended their own reference charts for diagnosing childhood obesity, as introduced by the Group of China Obesity Task Force in 2004 (COTF criteria) and the Centers for Disease Control and Prevention of the US in 2000 (CDC criteria) [10,15].

In 1993, the Hong Kong Growth Survey (HKGS) reported a sex-specific reference chart of weight-for-height to diagnose childhood obesity in Hong Kong (HKGS criteria) [16]. According to the HKGS, childhood obesity was defined as weight > [median weight-for-height × 120%]. Based on this chart, the obesity rates in our survey were 11–27% in boys and 12–17% in girls. These figures were much higher than those derived using other criteria such as the IOTF or COTF criteria, which gave a rate of less than 10% in almost all age groups of either sex (except boys aged 11 years). The HKGS criteria were reported more than 15 years ago and given the rapid changes in lifestyle, might not be applicable to today's young population. Besides, the HKGS criteria uses median body weight while BMI is now recommended for screening overweight/obesity in children and adolescents [11,14,18]. These discrepant findings also highlight the importance of periodically reviewing the national growth charts. However, it also has to be borne in mid that constantly redefining obestiy based on contemporary measures will make comparisons of secular data very difficult.

The IOTF criteria using an international BMI-for-age reference curve are widely frequently adopted by different countries to allow international comparisons of prevalence rates of obesity in children. Based on the IOFT criteria, the overall crude rate of obesity in Hong Kong children aged 11–18 years was 2.8% with a higher rate in boys than girls (3.9% vs. 1.8%). Although this rate was considerably lower than that reported in the US (10.9% in 1996 and 22.1% in 2001) and Europe (9–29%) [19,20], it is noteworthy that the younger the age, the higher the obesity rate, especially in boys. This is in accord to other reports that the prevalence usually peaks in those who were younger [21]. Irrespective of the criteria, 10–20% of our Chinese boys aged 11–12 years were considered obese compared to only 2 to 3% among those aged 16–18 years. The exact reasons behind are not clear but likely to be multi-factorial including early pubertal development in those Chinese children at ages 10–13 years. On the other hand, the obesity rate by IOTF criteria was lower than the rate by COTF criteria. This is not unexpected since the cutoff values for obesity is 30 kg/m^2 ^in the former as compared to 28 kg/m^2 ^in the latter.

Obesity is now a global concern among adults as well as children and adolescents. A significant proportion of obese children will eventually become obese adults [22]. Childhood obesity is associated with an increased rate of mortality and morbidity in adulthood [23]. Woo *et al *studied 36 asymptomatic overweight Chinese children (BMI > 23 kg/m^2^, aged 9–12 year) and 36 age- and gender-matched non-obese healthy children (BMI < 21 kg/m^2^) in Hong Kong [24]. The two groups of children were well matched for blood pressures, blood cholesterol and blood glucose levels. The overweight children, as compared to non-obese counter-parts, were found to have impaired arterial endothelial function and increased intima-medial thickness of carotid arteries. These findings highlight the potential impact of overweight, even of mild-to-moderate degree, on arterial function and structure in apparently healthy young children.

According to a survey conducted by the Hong Kong Children Health Service, the obesity rates (defined by the HKGS criteria) of Hong Kong primary and secondary school student boys and girls increased from 12.7% and 10.4% in 1998 to 14.7% and 12.4%, respectively, in 2001 [25]. Our data showed a further increase of obesity rate to 15.9% (boys: 18.1%; girls: 13.9%) in Hong Kong adolescents in 2004.

Relevant figures using other criteria on childhood obesity is lacking in Hong Kong. The present analysis reported the most recent prevalence of overweight and obesity in Hong Kong adolescents. A comparison of data at different time intervals based on these criteria is not available. However, it cannot be overemphasized that studying the trend of change of obesity rates is important in different regions of the world including our locality with Chinese populations.

## Conclusion

Our findings indicated that the prevalence rates of obesity in Hong Kong adolescents using various diagnostic criteria were similar except for the 1993 HKGS criteria, which gave an exceedingly high figure. Using the more recently proposed IOTF, COTF or CDC criteria, the prevalence of adolescent obesity in Hong Kong varied from 2.8% to 4.8% (3.9–6.0% in boys and 2.4–3.7% in girls) with particularly high rate in young boys. Although the present figures appear to be lower than that reported in Caucasians, BMI was the main determinant for metabolic syndrome reported to be 2–3% in these adolescents [26]. Given their high rates of obesity, pre-adolescents are an important population for monitoring and intervention.

## Abbreviations

BMI: Body mass index; IOTF: International Obesity Task Force; COTF: China Obesity Task Force; CDC: Centers for Disease Control and Prevention; HKGS: Hong Kong Growth Survey.

## Competing interests

The author(s) declare that they have no competing interests.

## Authors' contributions

GK did the data analyses, manuscript preparation and revision. GW, PT and JC critically reviewed the analyses and the manuscript. RO, AK and WS participated in the data collection and provided suggestions for manuscript revision. MC, CH and CL provided suggestions for analyses and manuscript revision. All authors read and approved the final manuscript.

## Pre-publication history

The pre-publication history for this paper can be accessed here:



## References

[B1] World Health Organization (2000). Obesity: Preventing and managing the global epidemic. Report of a WHO consultations. WHO Technical Report Series, No 894: Geneva.

[B2] Swallen KC, Reither EN, Haas SA, Meier AM (2005). Overweight, obesity, and health-related quality of life among adolescents: the National Longitudinal Study of Adolescent Health. Pediatrics.

[B3] Reilly JJ, Wilson D (2006). ABC of obesity. Childhood obesity. Br Med J.

[B4] Vanhala M, Vanhala P, Kumpusalo E, Halonen P, Takala J (1998). Relation between obesity from childhood to adulthood and the metabolic syndrome: population based study. Br Med J.

[B5] Rudolf MC, Sahota P, Barth JH, Walker J (2001). Increasing prevalence of obesity in primary school children: cohort study. Br Med J.

[B6] Burke V (2006). Obesity in childhood and cardiovascular risk. Clin Exp Pharmacol Physiol.

[B7] Karnehed N, Rasmussen F, Kark M (2007). Obesity in young adulthood and later disability pension: a population-based cohort study of 366,929 Swedish men. Scand J Public Health.

[B8] WHO Obesity and overweight. http://www.who.int/mediacentre/factsheets/fs311/en/.

[B9] Zhou BF (2002). Cooperative Meta-Analysis Group of the Working Group on Obesity in China. Predictive values of body mass index and waist circumference for risk factors of certain related diseases in Chinese adults – study on optimal cut-off points of body mass index and waist circumference in Chinese adults. Biomed Environ Sci.

[B10] Group of China Obesity Task Force (2004). Body mass index reference norm for screening overweight and obesity in Chinese children and adolescents. Chin J Epidemiol.

[B11] Cole TJ, Bellizzi MC, Flegal KM, Dietz WH (2000). Establishing a standard definition for child overweight and obesity worldwide: international survey. Br Med J.

[B12] Power C, Lake JK, Cole TJ (1997). Measurement and long-term health risks of child and adolescent fatness. Int J Obes Relat Metab Disord.

[B13] Reilly JJ (2002). Assessment of childhood obesity: national reference data or international approach?. Obes Res.

[B14] Dietz WH, Robinson TN (1998). Use of the body mass index (BMI) as a measure of overweight in children and adolescents. J Pediatr.

[B15] Ogden CL, Kuczmarski RJ, Flegal KM, Mei Z, Guo S, Wei R, Grummer-Strawn LM, Curtin LR, Roche AF, Johnson CL (2002). Centers for Disease Control and Prevention 2000 growth charts for the United States: improvements to the 1977 National Center for Health Statistics version. Pediatrics.

[B16] Leung SS, Lau JT, Tse LY, Oppenheimer SJ (1996). Weight-for-age and weight-for-height references for Hong Kong children from birth to 18 years. J Paediatr Child Health.

[B17] Bray GA (2004). Medical consequences of obesity. J Clin Endocrinol Metab.

[B18] Himes JH, Dietz WH (1994). Guidelines for overweight in adolescent preventive services: recommendations from an expert committee. The Expert Committee on Clinical Guidelines for Overweight in Adolescent Preventive Services. Am J Clin Nutr.

[B19] Gordon-Larsen P, Adair LS, Nelson MC, Popkin BM (2004). Five-year obesity incidence in the transition period between adolescence and adulthood: the National Longitudinal Study of Adolescent Health. Am J Clin Nutr.

[B20] Lissau I (2004). Overweight and obesity epidemic among children. Answer from European countries. Int J Obes Relat Metab Disord.

[B21] Rappaport EB, Robbins JM (2005). Overweight in Southeastern Pennsylvania children: 2002 household health survey data. Public Health Rep.

[B22] Vanhala M, Vanhala P, Kumpusalo E, Halonen P, Takala J (1998). Relation between obesity from childhood to adulthood and the metabolic syndrome: population based study. Br Med J.

[B23] Rudolf MC, Sahota P, Barth JH, Walker J (2001). Increasing prevalence of obesity in primary school children: cohort study. Br Med J.

[B24] Woo KS, Chook P, Yu CW, RY, Qiao M, Leung SS, Lam CW, Metreweli C, Celermajer DS (2004). Overweight in children is associated with arterial endothelial dysfunction and intima-media thickening. Int J Obes Relat Metab Disord.

[B25] Student Health Service, Department of Health, The Government of the Hong Kong Special Administrative Region. http://www.studenthealth.gov.hk/english/related/related.html/.

[B26] Ozaki R, Qiao Q, Wong GW, Chan MH, So WY, Tong PC, Ho CS, Ko GT, Kong AP, Lam CW, Tuomilehto J, Chan JC (2007). Overweight, family history of diabetes and attending schools of lower academic grading are independent predictors for metabolic syndrome in Hong Kong Chinese adolescents. Arch Dis Child.

